# Supplementary material for: SARS-CoV-2 clade dynamics and their associations with hospitalisations during the first two years of the COVID-19 pandemic
Source: PLoS One. 2024 May 10;19(5):e0303176. doi: 10.1371/journal.pone.0303176 (PMC11086870; doi:10.1371/journal.pone.0303176)
Supplement: S1 Table — Epidemiological waves were defined by the Estonian Health Board. Values are counts and percentages relative to the column are shown in parentheses. (DOCX) [file pone.0303176.s001.docx]

| **Lineage** | **N** | **Overall** | **1st wave (2020/02/26-2020/06/30)** | **2nd wave (2020/09/01-2021/06/30)** | **3rd wave (2021/07/01-2021/12/12)** | **4th wave (2021/12/13-2022/03/31)** |
| --- | --- | --- | --- | --- | --- | --- |
| **Delta, n (%)** | 10,744 |  |  |  |  |  |
| AY.122 |  | 4,589 (43) | 0 (NA) | 307 (88) | 3,913 (42) | 369 (35) |
| AY.100 |  | 1,102 (10) | 0 (NA) | 0 (0) | 1,059 (11) | 43 (4) |
| AY.43 |  | 789 (7) | 0 (NA) | 0 (0) | 679 (7) | 110 (10) |
| AY.122.2 |  | 702 (7) | 0 (NA) | 0 (0) | 688 (7) | 14 (1) |
| AY.4.5 |  | 474 (4) | 0 (NA) | 0 (0) | 436 (5) | 38 (4) |
| B.1.617.2 |  | 457 (4) | 0 (NA) | 27 (8) | 395 (4) | 35 (3) |
| AY.4 |  | 422 (4) | 0 (NA) | 6 (2) | 354 (4) | 62 (6) |
| AY.9.2 |  | 417 (4) | 0 (NA) | 0 (0) | 406 (4) | 11 (1) |
| AY.128 |  | 405 (4) | 0 (NA) | 0 (0) | 340 (4) | 65 (6) |
| AY.121 |  | 240 (2) | 0 (NA) | 0 (0) | 182 (2) | 58 (6) |
| AY.33 |  | 196 (2) | 0 (NA) | 0 (0) | 185 (2) | 11 (1) |
| AY.46 |  | 175 (2) | 0 (NA) | 0 (0) | 138 (1) | 37 (4) |
| AY.127 |  | 164 (2) | 0 (NA) | 0 (0) | 68 (1) | 96 (9) |
| AY.126 |  | 109 (1) | 0 (NA) | 1 (0) | 81 (1) | 27 (3) |
| AY.5 |  | 46 (0) | 0 (NA) | 2 (1) | 43 (0) | 1 (0) |
| AY.98.1 |  | 43 (0) | 0 (NA) | 0 (0) | 37 (0) | 6 (1) |
| AY.4.2 |  | 34 (0) | 0 (NA) | 0 (0) | 22 (0) | 12 (1) |
| AY.125 |  | 26 (0) | 0 (NA) | 0 (0) | 23 (0) | 3 (0) |
| AY.3 |  | 26 (0) | 0 (NA) | 0 (0) | 26 (0) | 0 (0) |
| AY.36 |  | 25 (0) | 0 (NA) | 0 (0) | 20 (0) | 5 (0) |
| AY.6 |  | 20 (0) | 0 (NA) | 0 (0) | 19 (0) | 1 (0) |
| AY.68 |  | 20 (0) | 0 (NA) | 0 (0) | 20 (0) | 0 (0) |
| AY.124 |  | 19 (0) | 0 (NA) | 0 (0) | 18 (0) | 1 (0) |
| AY.42 |  | 18 (0) | 0 (NA) | 0 (0) | 18 (0) | 0 (0) |
| AY.4.4 |  | 17 (0) | 0 (NA) | 0 (0) | 17 (0) | 0 (0) |
| AY.4.2.3 |  | 13 (0) | 0 (NA) | 0 (0) | 4 (0) | 9 (1) |
| AY.7.1 |  | 13 (0) | 0 (NA) | 0 (0) | 13 (0) | 0 (0) |
| AY.70 |  | 13 (0) | 0 (NA) | 0 (0) | 13 (0) | 0 (0) |
| AY.46.6 |  | 11 (0) | 0 (NA) | 0 (0) | 10 (0) | 1 (0) |
| AY.25.1 |  | 10 (0) | 0 (NA) | 0 (0) | 5 (0) | 5 (0) |
| AY.36.1 |  | 10 (0) | 0 (NA) | 0 (0) | 6 (0) | 4 (0) |
| AY.103 |  | 8 (0) | 0 (NA) | 0 (0) | 6 (0) | 2 (0) |
| AY.46.5 |  | 8 (0) | 0 (NA) | 0 (0) | 8 (0) | 0 (0) |
| AY.129 |  | 7 (0) | 0 (NA) | 0 (0) | 7 (0) | 0 (0) |
| AY.120 |  | 6 (0) | 0 (NA) | 0 (0) | 6 (0) | 0 (0) |
| AY.54 |  | 6 (0) | 0 (NA) | 0 (0) | 6 (0) | 0 (0) |
| AY.106 |  | 5 (0) | 0 (NA) | 0 (0) | 1 (0) | 4 (0) |
| AY.121.1 |  | 5 (0) | 0 (NA) | 0 (0) | 5 (0) | 0 (0) |
| AY.23 |  | 5 (0) | 0 (NA) | 0 (0) | 5 (0) | 0 (0) |
| AY.34 |  | 5 (0) | 0 (NA) | 0 (0) | 4 (0) | 1 (0) |
| AY.4.6 |  | 5 (0) | 0 (NA) | 0 (0) | 5 (0) | 0 (0) |
| AY.118 |  | 4 (0) | 0 (NA) | 0 (0) | 4 (0) | 0 (0) |
| AY.4.7 |  | 4 (0) | 0 (NA) | 0 (0) | 4 (0) | 0 (0) |
| AY.4.9 |  | 4 (0) | 0 (NA) | 0 (0) | 4 (0) | 0 (0) |
| AY.98 |  | 4 (0) | 0 (NA) | 0 (0) | 1 (0) | 3 (0) |
| AY.112 |  | 3 (0) | 0 (NA) | 0 (0) | 3 (0) | 0 (0) |
| AY.4.2.1 |  | 3 (0) | 0 (NA) | 0 (0) | 1 (0) | 2 (0) |
| AY.53 |  | 3 (0) | 0 (NA) | 0 (0) | 3 (0) | 0 (0) |
| AY.60 |  | 3 (0) | 0 (NA) | 0 (0) | 3 (0) | 0 (0) |
| AY.7.2 |  | 3 (0) | 0 (NA) | 0 (0) | 2 (0) | 1 (0) |
| AY.78 |  | 3 (0) | 0 (NA) | 0 (0) | 3 (0) | 0 (0) |
| AY.9.2.2 |  | 3 (0) | 0 (NA) | 0 (0) | 0 (0) | 3 (0) |
| AY.16 |  | 2 (0) | 0 (NA) | 2 (1) | 0 (0) | 0 (0) |
| AY.25 |  | 2 (0) | 0 (NA) | 0 (0) | 2 (0) | 0 (0) |
| AY.28 |  | 2 (0) | 0 (NA) | 0 (0) | 0 (0) | 2 (0) |
| AY.29 |  | 2 (0) | 0 (NA) | 0 (0) | 2 (0) | 0 (0) |
| AY.4.2.2 |  | 2 (0) | 0 (NA) | 0 (0) | 2 (0) | 0 (0) |
| AY.4.3 |  | 2 (0) | 0 (NA) | 0 (0) | 2 (0) | 0 (0) |
| AY.43.3 |  | 2 (0) | 0 (NA) | 0 (0) | 2 (0) | 0 (0) |
| AY.43.6 |  | 2 (0) | 0 (NA) | 0 (0) | 1 (0) | 1 (0) |
| AY.43.8 |  | 2 (0) | 0 (NA) | 0 (0) | 2 (0) | 0 (0) |
| AY.46.2 |  | 2 (0) | 0 (NA) | 0 (0) | 2 (0) | 0 (0) |
| AY.47 |  | 2 (0) | 0 (NA) | 0 (0) | 2 (0) | 0 (0) |
| AY.95 |  | 2 (0) | 0 (NA) | 1 (0) | 1 (0) | 0 (0) |
| AY.99.2 |  | 2 (0) | 0 (NA) | 0 (0) | 0 (0) | 2 (0) |
| AY.108 |  | 1 (0) | 0 (NA) | 0 (0) | 1 (0) | 0 (0) |
| AY.111 |  | 1 (0) | 0 (NA) | 0 (0) | 0 (0) | 1 (0) |
| AY.113 |  | 1 (0) | 0 (NA) | 0 (0) | 1 (0) | 0 (0) |
| AY.116 |  | 1 (0) | 0 (NA) | 1 (0) | 0 (0) | 0 (0) |
| AY.119 |  | 1 (0) | 0 (NA) | 0 (0) | 1 (0) | 0 (0) |
| AY.120.2.1 |  | 1 (0) | 0 (NA) | 0 (0) | 1 (0) | 0 (0) |
| AY.13 |  | 1 (0) | 0 (NA) | 0 (0) | 1 (0) | 0 (0) |
| AY.3.1 |  | 1 (0) | 0 (NA) | 0 (0) | 1 (0) | 0 (0) |
| AY.35 |  | 1 (0) | 0 (NA) | 0 (0) | 1 (0) | 0 (0) |
| AY.38 |  | 1 (0) | 0 (NA) | 0 (0) | 1 (0) | 0 (0) |
| AY.4.11 |  | 1 (0) | 0 (NA) | 0 (0) | 0 (0) | 1 (0) |
| AY.4.15 |  | 1 (0) | 0 (NA) | 0 (0) | 1 (0) | 0 (0) |
| AY.43.2 |  | 1 (0) | 0 (NA) | 0 (0) | 1 (0) | 0 (0) |
| AY.43.4 |  | 1 (0) | 0 (NA) | 0 (0) | 0 (0) | 1 (0) |
| AY.44 |  | 1 (0) | 0 (NA) | 0 (0) | 1 (0) | 0 (0) |
| AY.55 |  | 1 (0) | 0 (NA) | 0 (0) | 1 (0) | 0 (0) |
| Unknown |  | 49 | 0 | 10 | 38 | 1 |
| **Omicron, n (%)** | 6,803 |  |  |  |  |  |
| BA.2 |  | 2,185 (32) | 0 (NA) | 0 (NA) | 0 (0) | 2,185 (32) |
| BA.1.1 |  | 2,080 (31) | 0 (NA) | 0 (NA) | 5 (15) | 2,075 (31) |
| BA.1 |  | 942 (14) | 0 (NA) | 0 (NA) | 20 (59) | 922 (14) |
| BA.2.9 |  | 783 (12) | 0 (NA) | 0 (NA) | 0 (0) | 783 (12) |
| BA.1.17.2 |  | 170 (2) | 0 (NA) | 0 (NA) | 3 (9) | 167 (2) |
| BA.1.17 |  | 108 (2) | 0 (NA) | 0 (NA) | 2 (6) | 106 (2) |
| BA.1.15 |  | 98 (1) | 0 (NA) | 0 (NA) | 1 (3) | 97 (1) |
| BA.1.1.1 |  | 96 (1) | 0 (NA) | 0 (NA) | 0 (0) | 96 (1) |
| BA.1.18 |  | 52 (1) | 0 (NA) | 0 (NA) | 0 (0) | 52 (1) |
| BA.1.15.1 |  | 49 (1) | 0 (NA) | 0 (NA) | 0 (0) | 49 (1) |
| BA.2.3 |  | 40 (1) | 0 (NA) | 0 (NA) | 0 (0) | 40 (1) |
| B.1.1.529 |  | 20 (0) | 0 (NA) | 0 (NA) | 2 (6) | 18 (0) |
| BA.1.16 |  | 18 (0) | 0 (NA) | 0 (NA) | 0 (0) | 18 (0) |
| BA.1.8 |  | 18 (0) | 0 (NA) | 0 (NA) | 0 (0) | 18 (0) |
| BA.1.14 |  | 16 (0) | 0 (NA) | 0 (NA) | 0 (0) | 16 (0) |
| BA.1.1.11 |  | 13 (0) | 0 (NA) | 0 (NA) | 0 (0) | 13 (0) |
| BA.1.20 |  | 13 (0) | 0 (NA) | 0 (NA) | 0 (0) | 13 (0) |
| BA.1.13 |  | 12 (0) | 0 (NA) | 0 (NA) | 0 (0) | 12 (0) |
| BA.1.21 |  | 8 (0) | 0 (NA) | 0 (NA) | 1 (3) | 7 (0) |
| BA.1.1.13 |  | 7 (0) | 0 (NA) | 0 (NA) | 0 (0) | 7 (0) |
| BA.1.1.15 |  | 7 (0) | 0 (NA) | 0 (NA) | 0 (0) | 7 (0) |
| BA.1.19 |  | 7 (0) | 0 (NA) | 0 (NA) | 0 (0) | 7 (0) |
| BA.1.9 |  | 7 (0) | 0 (NA) | 0 (NA) | 0 (0) | 7 (0) |
| BA.2.23 |  | 7 (0) | 0 (NA) | 0 (NA) | 0 (0) | 7 (0) |
| BA.2.25 |  | 7 (0) | 0 (NA) | 0 (NA) | 0 (0) | 7 (0) |
| BA.2.9.5 |  | 7 (0) | 0 (NA) | 0 (NA) | 0 (0) | 7 (0) |
| BA.1.1.18 |  | 6 (0) | 0 (NA) | 0 (NA) | 0 (0) | 6 (0) |
| BA.2.10 |  | 5 (0) | 0 (NA) | 0 (NA) | 0 (0) | 5 (0) |
| BA.2.9.2 |  | 3 (0) | 0 (NA) | 0 (NA) | 0 (0) | 3 (0) |
| BA.1.1.14 |  | 2 (0) | 0 (NA) | 0 (NA) | 0 (0) | 2 (0) |
| BA.1.1.7 |  | 2 (0) | 0 (NA) | 0 (NA) | 0 (0) | 2 (0) |
| BA.1.12 |  | 2 (0) | 0 (NA) | 0 (NA) | 0 (0) | 2 (0) |
| BA.2.45 |  | 2 (0) | 0 (NA) | 0 (NA) | 0 (0) | 2 (0) |
| BA.2.51 |  | 2 (0) | 0 (NA) | 0 (NA) | 0 (0) | 2 (0) |
| BA.1.1.2 |  | 1 (0) | 0 (NA) | 0 (NA) | 0 (0) | 1 (0) |
| BA.1.14.2 |  | 1 (0) | 0 (NA) | 0 (NA) | 0 (0) | 1 (0) |
| BA.1.21.1 |  | 1 (0) | 0 (NA) | 0 (NA) | 0 (0) | 1 (0) |
| BA.1.6 |  | 1 (0) | 0 (NA) | 0 (NA) | 0 (0) | 1 (0) |
| BA.1.7 |  | 1 (0) | 0 (NA) | 0 (NA) | 0 (0) | 1 (0) |
| BA.2.1 |  | 1 (0) | 0 (NA) | 0 (NA) | 0 (0) | 1 (0) |
| BA.2.19 |  | 1 (0) | 0 (NA) | 0 (NA) | 0 (0) | 1 (0) |
| BA.2.7 |  | 1 (0) | 0 (NA) | 0 (NA) | 0 (0) | 1 (0) |
| BA.2.8 |  | 1 (0) | 0 (NA) | 0 (NA) | 0 (0) | 1 (0) |
| Unknown |  | 48 | 0 | 0 | 0 | 48 |
| **Alpha, n (%)** | 5,795 |  |  |  |  |  |
| B.1.1.7 |  | 5,747 (99) | 0 (NA) | 5,699 (99) | 48 (100) | 0 (NA) |
| Q.4 |  | 38 (1) | 0 (NA) | 38 (1) | 0 (0) | 0 (NA) |
| Q.1 |  | 8 (0) | 0 (NA) | 8 (0) | 0 (0) | 0 (NA) |
| Q.7 |  | 1 (0) | 0 (NA) | 1 (0) | 0 (0) | 0 (NA) |
| Q.8 |  | 1 (0) | 0 (NA) | 1 (0) | 0 (0) | 0 (NA) |
| Unknown |  | 47 | 0 | 47 | 0 | 0 |
| **Beta, n (%)** | 73 |  |  |  |  |  |
| B.1.351 |  | 73 (100) | 0 (NA) | 73 (100) | 0 (NA) | 0 (NA) |
| **Gamma, n (%)** | 10 |  |  |  |  |  |
| P.1 |  | 5 (50) | 0 (NA) | 1 (17) | 4 (100) | 0 (NA) |
| P.1.16 |  | 5 (50) | 0 (NA) | 5 (83) | 0 (0) | 0 (NA) |
| **Other, n (%)** | 1,750 |  |  |  |  |  |
| B.1.177.60 |  | 288 (16) | 0 (0) | 288 (17) | 0 (0) | 0 (NA) |
| B.1.1 |  | 234 (13) | 15 (94) | 219 (13) | 0 (0) | 0 (NA) |
| B.1.258 |  | 216 (12) | 0 (0) | 216 (12) | 0 (0) | 0 (NA) |
| B.1.221 |  | 168 (10) | 0 (0) | 168 (10) | 0 (0) | 0 (NA) |
| B.1.1.317 |  | 163 (9) | 0 (0) | 163 (9) | 0 (0) | 0 (NA) |
| B.1.527 |  | 145 (8) | 0 (0) | 145 (8) | 0 (0) | 0 (NA) |
| B.1.1.397 |  | 77 (4) | 0 (0) | 77 (4) | 0 (0) | 0 (NA) |
| B.1.1.10 |  | 61 (3) | 0 (0) | 61 (4) | 0 (0) | 0 (NA) |
| B.1 |  | 53 (3) | 1 (6) | 52 (3) | 0 (0) | 0 (NA) |
| B.1.177 |  | 47 (3) | 0 (0) | 47 (3) | 0 (0) | 0 (NA) |
| C.36.3 |  | 40 (2) | 0 (0) | 40 (2) | 0 (0) | 0 (NA) |
| C.38 |  | 32 (2) | 0 (0) | 32 (2) | 0 (0) | 0 (NA) |
| B.1.160 |  | 28 (2) | 0 (0) | 28 (2) | 0 (0) | 0 (NA) |
| B.1.1.203 |  | 18 (1) | 0 (0) | 18 (1) | 0 (0) | 0 (NA) |
| B.1.1.1 |  | 16 (1) | 0 (0) | 16 (1) | 0 (0) | 0 (NA) |
| B.1.36 |  | 14 (1) | 0 (0) | 14 (1) | 0 (0) | 0 (NA) |
| B.1.1.159 |  | 13 (1) | 0 (0) | 13 (1) | 0 (0) | 0 (NA) |
| AT.1 |  | 11 (1) | 0 (0) | 11 (1) | 0 (0) | 0 (NA) |
| B.1.1.294 |  | 11 (1) | 0 (0) | 11 (1) | 0 (0) | 0 (NA) |
| B.1.1.523 |  | 10 (1) | 0 (0) | 10 (1) | 0 (0) | 0 (NA) |
| C.36 |  | 9 (1) | 0 (0) | 9 (1) | 0 (0) | 0 (NA) |
| A.18 |  | 7 (0) | 0 (0) | 7 (0) | 0 (0) | 0 (NA) |
| B.1.36.22 |  | 7 (0) | 0 (0) | 7 (0) | 0 (0) | 0 (NA) |
| B.1.1.318 |  | 6 (0) | 0 (0) | 6 (0) | 0 (0) | 0 (NA) |
| B.1.1.351 |  | 5 (0) | 0 (0) | 5 (0) | 0 (0) | 0 (NA) |
| B.1.1.374 |  | 4 (0) | 0 (0) | 4 (0) | 0 (0) | 0 (NA) |
| B.1.1.398 |  | 4 (0) | 0 (0) | 4 (0) | 0 (0) | 0 (NA) |
| B.1.466.1 |  | 4 (0) | 0 (0) | 4 (0) | 0 (0) | 0 (NA) |
| C.17 |  | 4 (0) | 0 (0) | 4 (0) | 0 (0) | 0 (NA) |
| AZ.2 |  | 3 (0) | 0 (0) | 3 (0) | 0 (0) | 0 (NA) |
| B.1.1.141 |  | 3 (0) | 0 (0) | 3 (0) | 0 (0) | 0 (NA) |
| B.1.1.519 |  | 3 (0) | 0 (0) | 3 (0) | 0 (0) | 0 (NA) |
| B.1.468 |  | 3 (0) | 0 (0) | 3 (0) | 0 (0) | 0 (NA) |
| B.1.1.216 |  | 2 (0) | 0 (0) | 2 (0) | 0 (0) | 0 (NA) |
| B.1.1.349 |  | 2 (0) | 0 (0) | 2 (0) | 0 (0) | 0 (NA) |
| B.1.1.51 |  | 2 (0) | 0 (0) | 2 (0) | 0 (0) | 0 (NA) |
| B.1.1.89 |  | 2 (0) | 0 (0) | 2 (0) | 0 (0) | 0 (NA) |
| B.1.177.21 |  | 2 (0) | 0 (0) | 2 (0) | 0 (0) | 0 (NA) |
| B.1.258.17 |  | 2 (0) | 0 (0) | 2 (0) | 0 (0) | 0 (NA) |
| B.1.525 |  | 2 (0) | 0 (0) | 2 (0) | 0 (0) | 0 (NA) |
| B.1.595 |  | 2 (0) | 0 (0) | 2 (0) | 0 (0) | 0 (NA) |
| A.2.5 |  | 1 (0) | 0 (0) | 1 (0) | 0 (0) | 0 (NA) |
| A.23.1 |  | 1 (0) | 0 (0) | 1 (0) | 0 (0) | 0 (NA) |
| B.1.1.153 |  | 1 (0) | 0 (0) | 1 (0) | 0 (0) | 0 (NA) |
| B.1.1.161 |  | 1 (0) | 0 (0) | 1 (0) | 0 (0) | 0 (NA) |
| B.1.1.241 |  | 1 (0) | 0 (0) | 1 (0) | 0 (0) | 0 (NA) |
| B.1.1.243 |  | 1 (0) | 0 (0) | 1 (0) | 0 (0) | 0 (NA) |
| B.1.1.307 |  | 1 (0) | 0 (0) | 1 (0) | 0 (0) | 0 (NA) |
| B.1.1.33 |  | 1 (0) | 0 (0) | 1 (0) | 0 (0) | 0 (NA) |
| B.1.1.372 |  | 1 (0) | 0 (0) | 1 (0) | 0 (0) | 0 (NA) |
| B.1.1.429 |  | 1 (0) | 0 (0) | 1 (0) | 0 (0) | 0 (NA) |
| B.1.1.524 |  | 1 (0) | 0 (0) | 1 (0) | 0 (0) | 0 (NA) |
| B.1.143 |  | 1 (0) | 0 (0) | 1 (0) | 0 (0) | 0 (NA) |
| B.1.177.81 |  | 1 (0) | 0 (0) | 1 (0) | 0 (0) | 0 (NA) |
| B.1.177.86 |  | 1 (0) | 0 (0) | 1 (0) | 0 (0) | 0 (NA) |
| B.1.214 |  | 1 (0) | 0 (0) | 1 (0) | 0 (0) | 0 (NA) |
| B.1.22 |  | 1 (0) | 0 (0) | 1 (0) | 0 (0) | 0 (NA) |
| B.1.356 |  | 1 (0) | 0 (0) | 1 (0) | 0 (0) | 0 (NA) |
| B.1.379 |  | 1 (0) | 0 (0) | 1 (0) | 0 (0) | 0 (NA) |
| B.1.396 |  | 1 (0) | 0 (0) | 1 (0) | 0 (0) | 0 (NA) |
| B.1.428 |  | 1 (0) | 0 (0) | 1 (0) | 0 (0) | 0 (NA) |
| B.1.617.1 |  | 1 (0) | 0 (0) | 1 (0) | 0 (0) | 0 (NA) |
| B.1.621 |  | 1 (0) | 0 (0) | 0 (0) | 1 (100) | 0 (NA) |
| B.1.637 |  | 1 (0) | 0 (0) | 1 (0) | 0 (0) | 0 (NA) |
| B.1.91 |  | 1 (0) | 0 (0) | 1 (0) | 0 (0) | 0 (NA) |
| C.14 |  | 1 (0) | 0 (0) | 1 (0) | 0 (0) | 0 (NA) |
| P.3 |  | 1 (0) | 0 (0) | 1 (0) | 0 (0) | 0 (NA) |
| S.1 |  | 1 (0) | 0 (0) | 1 (0) | 0 (0) | 0 (NA) |
| Unknown |  | 56 | 0 | 36 | 20 | 0 |
|  | | | | | | |
