# Supplementary figures and images for: SARS-CoV-2 clade dynamics and their associations with hospitalisations during the first two years of the COVID-19 pandemic
Source: PLoS One. 2024 May 10;19(5):e0303176. doi: 10.1371/journal.pone.0303176 (PMC11086870; doi:10.1371/journal.pone.0303176)

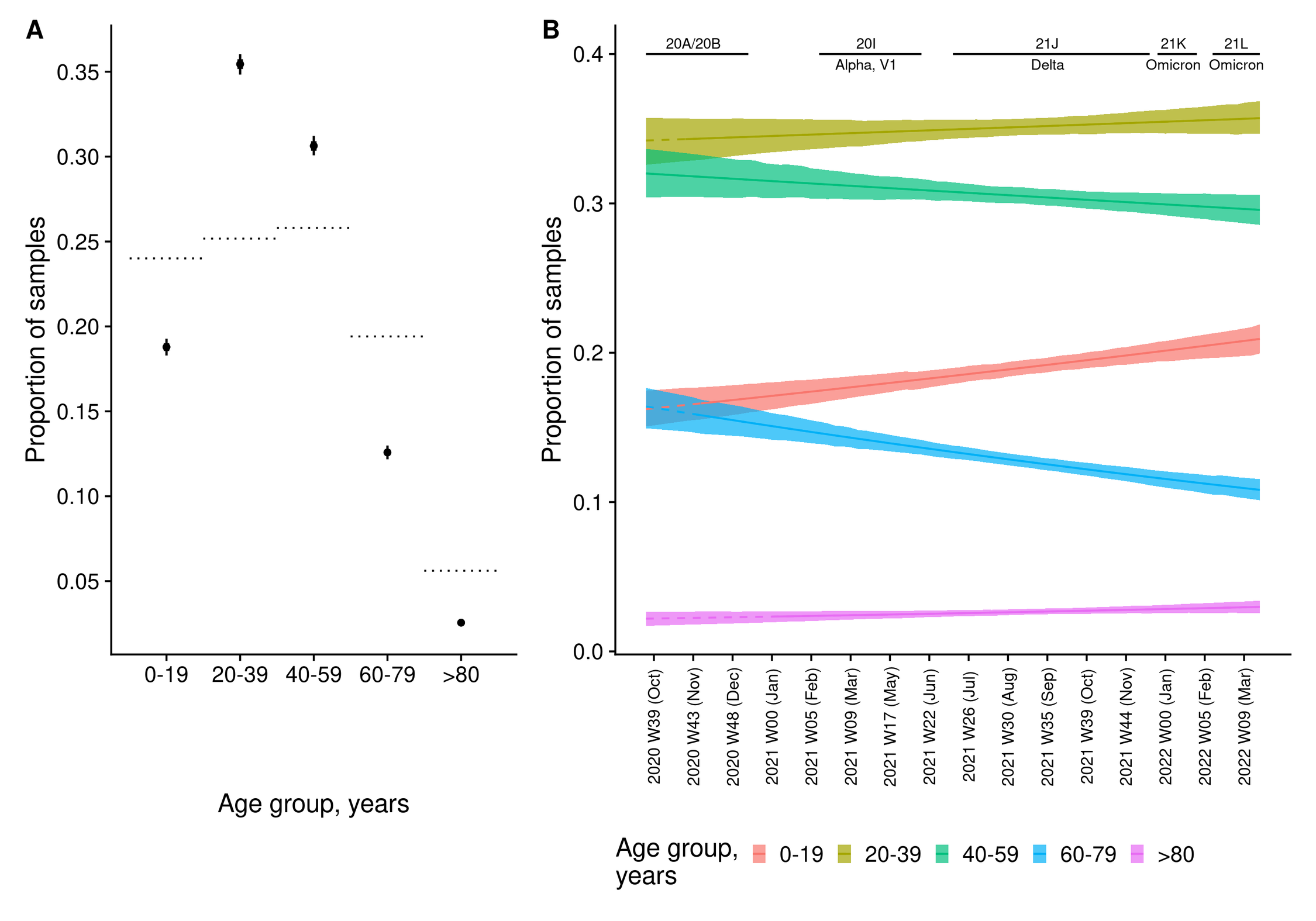

Supplement: S1 Fig — (A) the study population age group distribution relative to the 2022 general population. Points denote the best estimate of the aggregated binomial model, N = 26,618. Thick and thin lines denote 67% and 95% credible intervals, respectively. Dotted horizontal lines denote age group prevalence in the general population. (B) trends in the study population age group distribution during the study period. Lines denote the best estimate of the simple binomial model fitted to the weekly age group prevalence. Dashed lines denote unobserved dates implied by the underlying model. Ribbons denote a 95% credible interval. Solid horizontal lines denote SARS-Cov-2 waves. Panel A model summary is presented in Table 5 in S2 Appendix. Panel B model summary is presented in Table 6 in S2 Appendix. (TIF) [file pone.0303176.s002.tif]

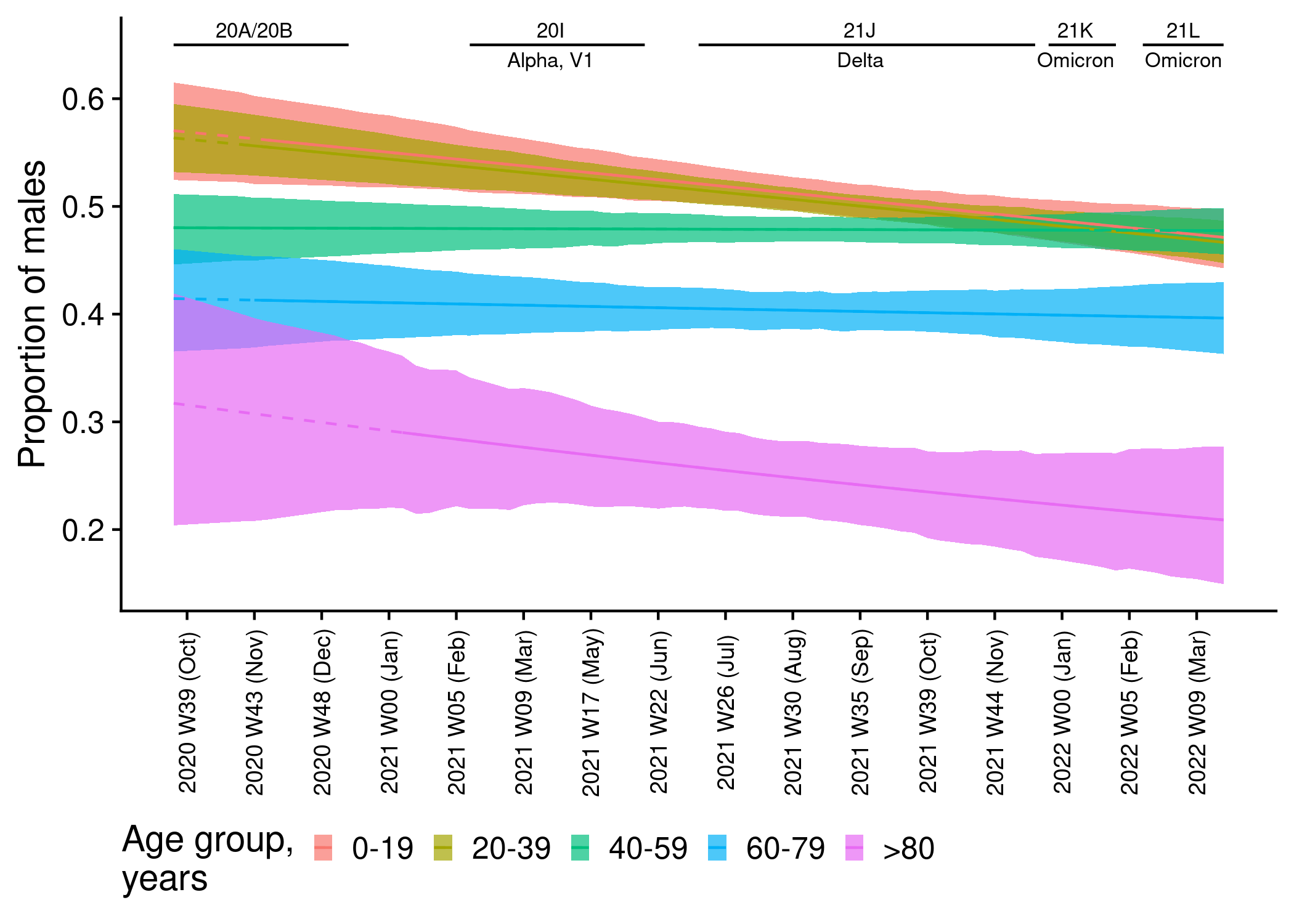

Supplement: S2 Fig — Lines denote the best estimate of the Bernoulli model, N = 24,343. The dashed lines denote unobserved dates implied by the underlying model. Ribbons denote a 95% credible interval. Solid horizontal lines denote SARS-Cov-2 waves. The model summary is presented in Table 7 in S2 Appendix. (TIF) [file pone.0303176.s003.tif]

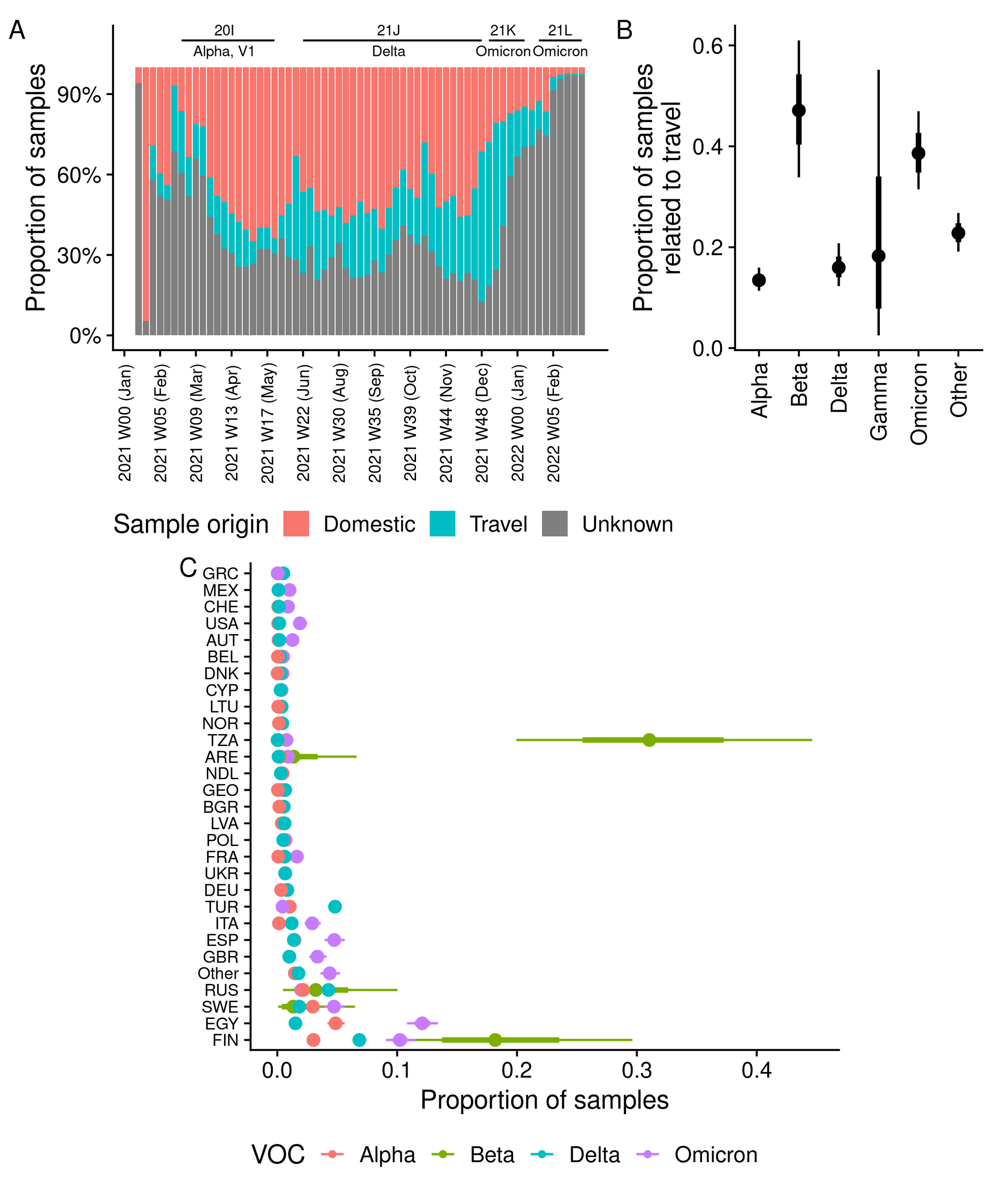

Supplement: S3 Fig — (A) the weekly proportions of domestic- and travel-related cases and cases where such info was missing. (B) the association of travel-related cases with VOC indicated by the Bernoulli model, adjusted for the proportion of cases with missing info and population vaccination coverage, N = 14,341. (C) VOC association with countries or territories of travel-related cases indicated by the aggregated binomial model, N = 13,603. Y-axis labels are country ISO codes, and “Other” includes countries or territories associated with less than 30 cases. In panels B and C, points denote the model’s best estimate, and thin and thick lines denote 66% and 95% credible intervals, respectively. Panel B model summary is presented in Table 8 in S2 Appendix. Panel C model summary is presented in Table 9 in S2 Appendix. (TIF) [file pone.0303176.s004.tif]

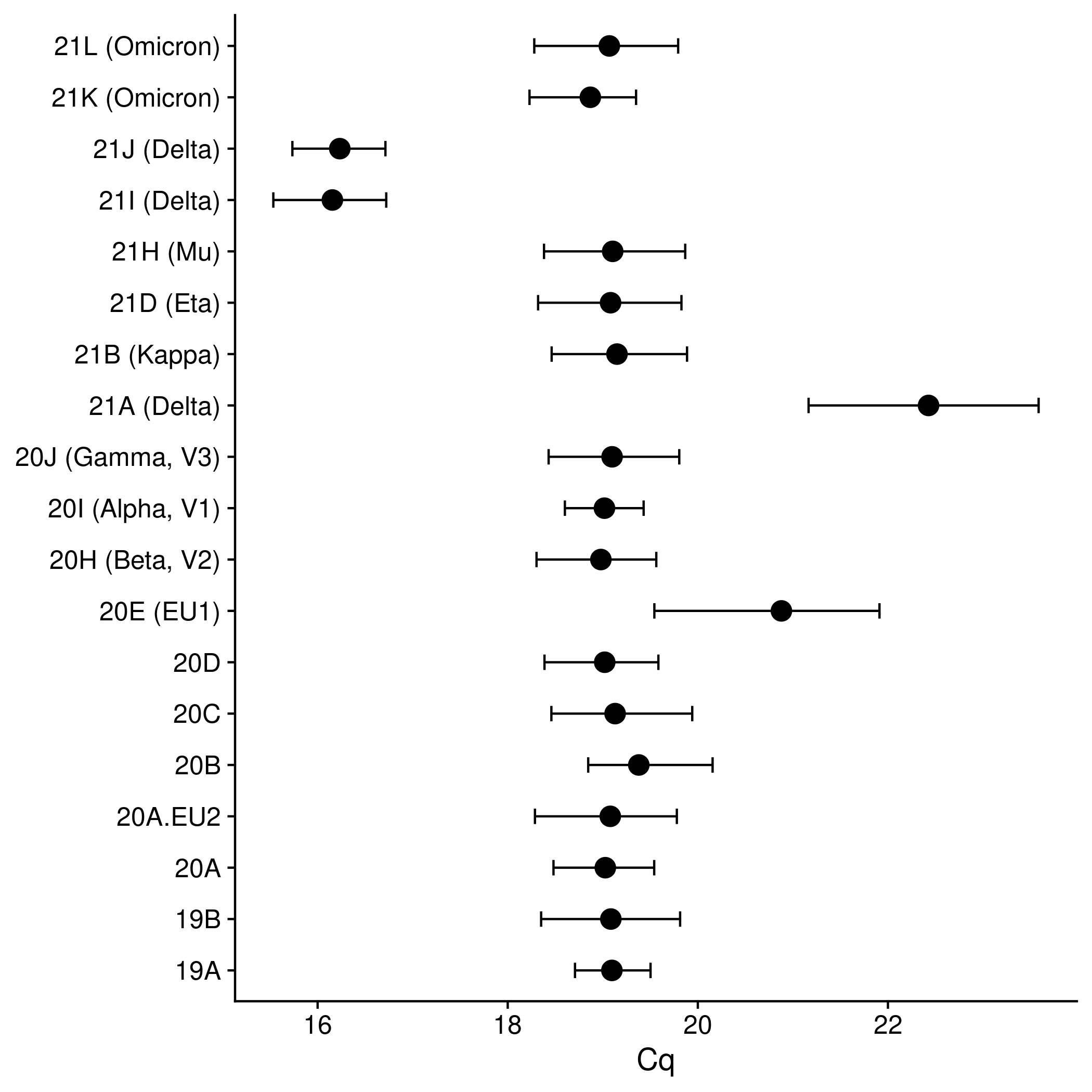

Supplement: S4 Fig — Posterior summaries of the average Cq value of ORF1a, S gene, and N gene, obtained from a linear model adjusted for age, sex, and vaccination status, skew-normal distribution, N = 5,689. S gene dropouts were treated as missing data and only ORF1a and N gene Ct values were used for these samples. Point denotes the model’s best fit and error bars denote a 95% credible interval. The model summary is presented in Table 10 in S2 Appendix. (TIF) [file pone.0303176.s005.tif]

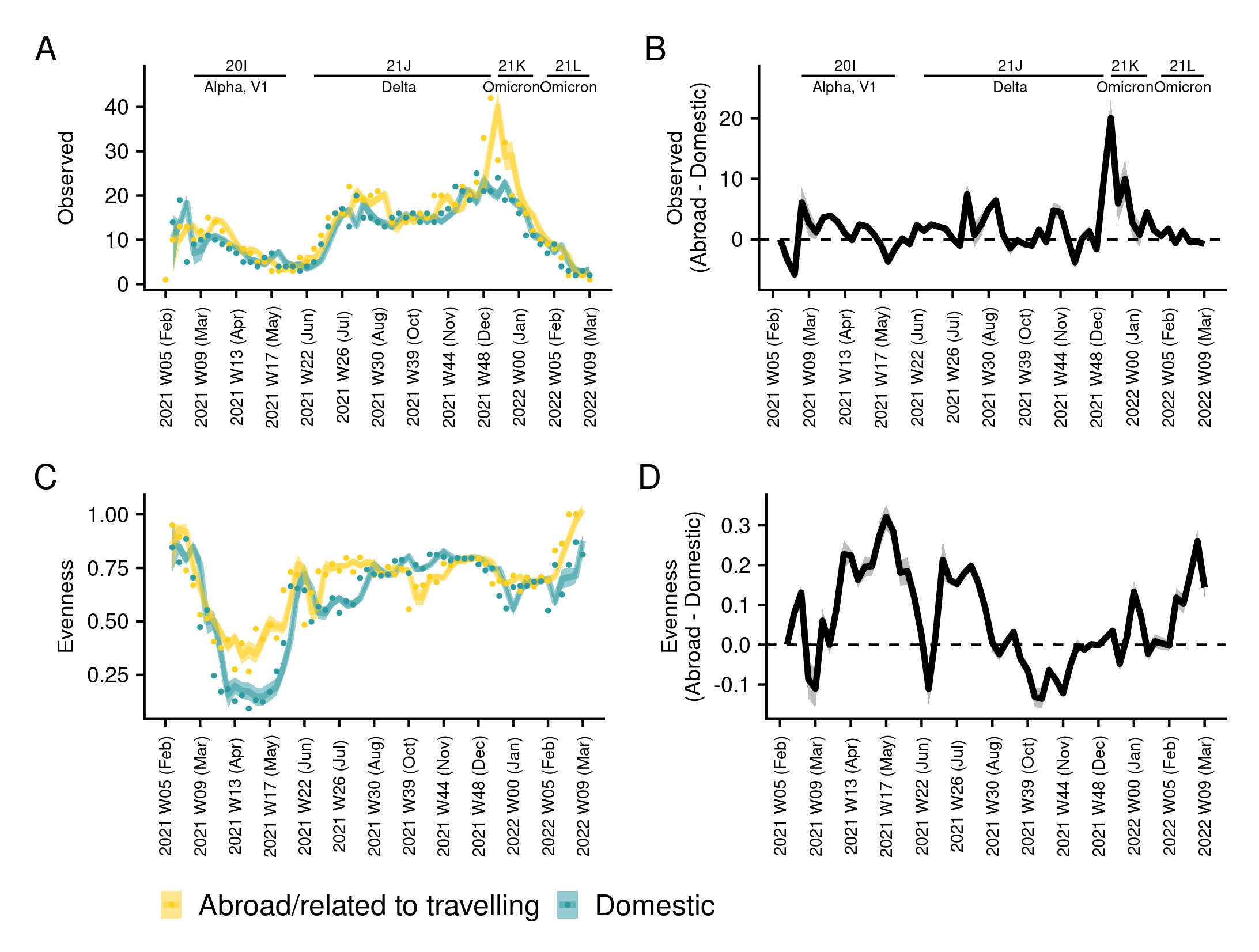

Supplement: S5 Fig — (A) the observed number of Pango lineages. (B) the difference in the observed number of Pango lineages, imported cases compared to domestic cases. (C) evenness index. (D) the effect size of the evenness index in imported cases compared to domestic cases. In panels A and C, points denote individual weekly observations. The line denotes the autoregressive model best fit and the shaded ribbon denotes a 95% credible interval. In panels B and D, the line denotes effect size derived from the model fit shown in panels A or C, respectively, and the shaded ribbon denotes a 95% credible interval. Panel A and B model summary is presented in Table 11 in S2 Appendix. Panel C and D model summary is presented in Table 12 in S2 Appendix. (TIF) [file pone.0303176.s006.tif]

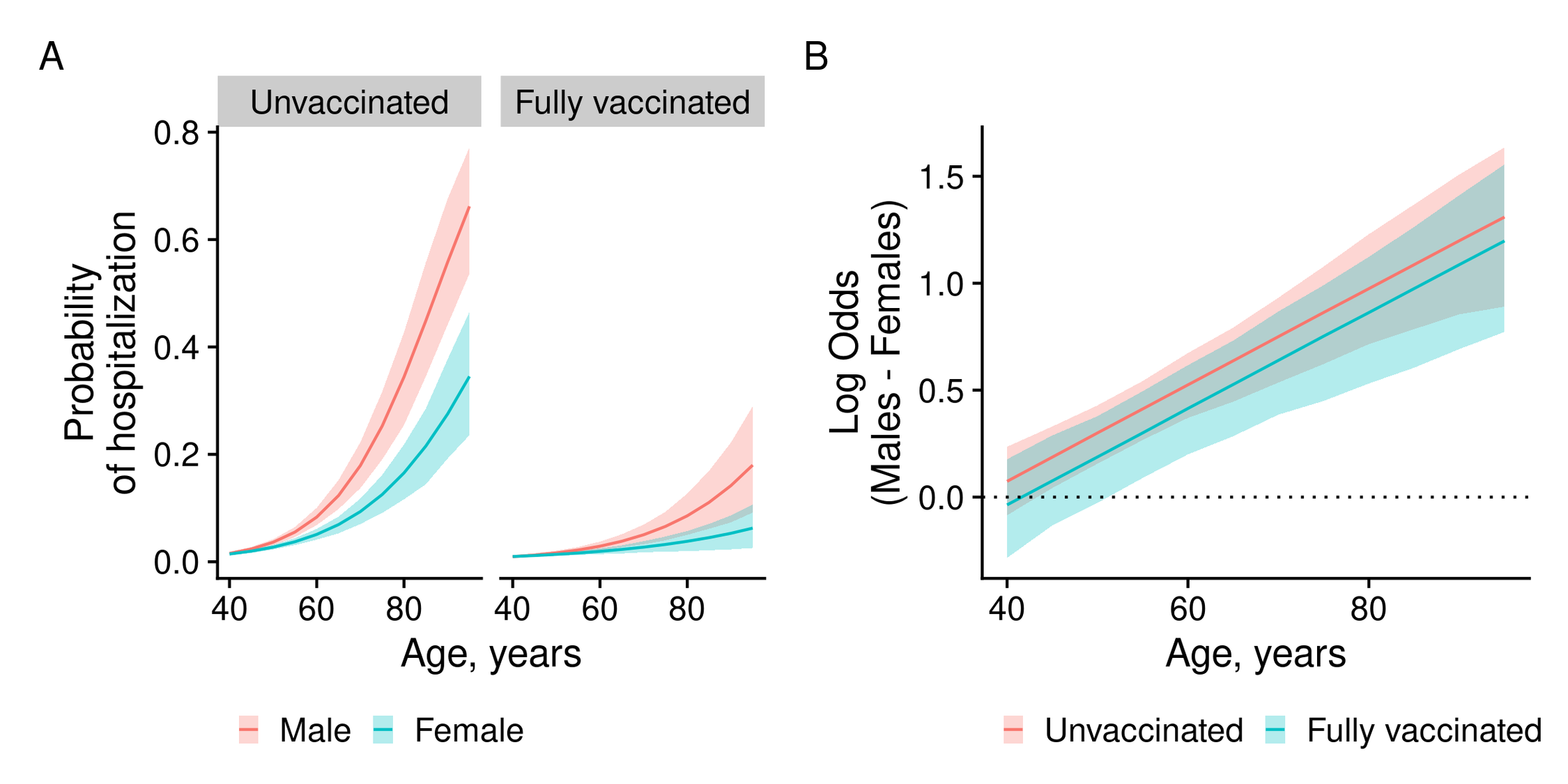

Supplement: S6 Fig — (A) the probability of hospitalization is associated with sex, age, and vaccination status. (B) the odds ratio of hospitalization of males relative to females is associated with age, but independent of vaccination status. Posterior distributions of the estimated marginal means were obtained from the same model as in Fig 5. The line denotes the model’s best estimate and the ribbon denotes a 95% credible interval. The dotted line denotes the log odds ratio = 0. The model summary is presented in Table 4 in S2 Appendix. (TIF) [file pone.0303176.s007.tif]

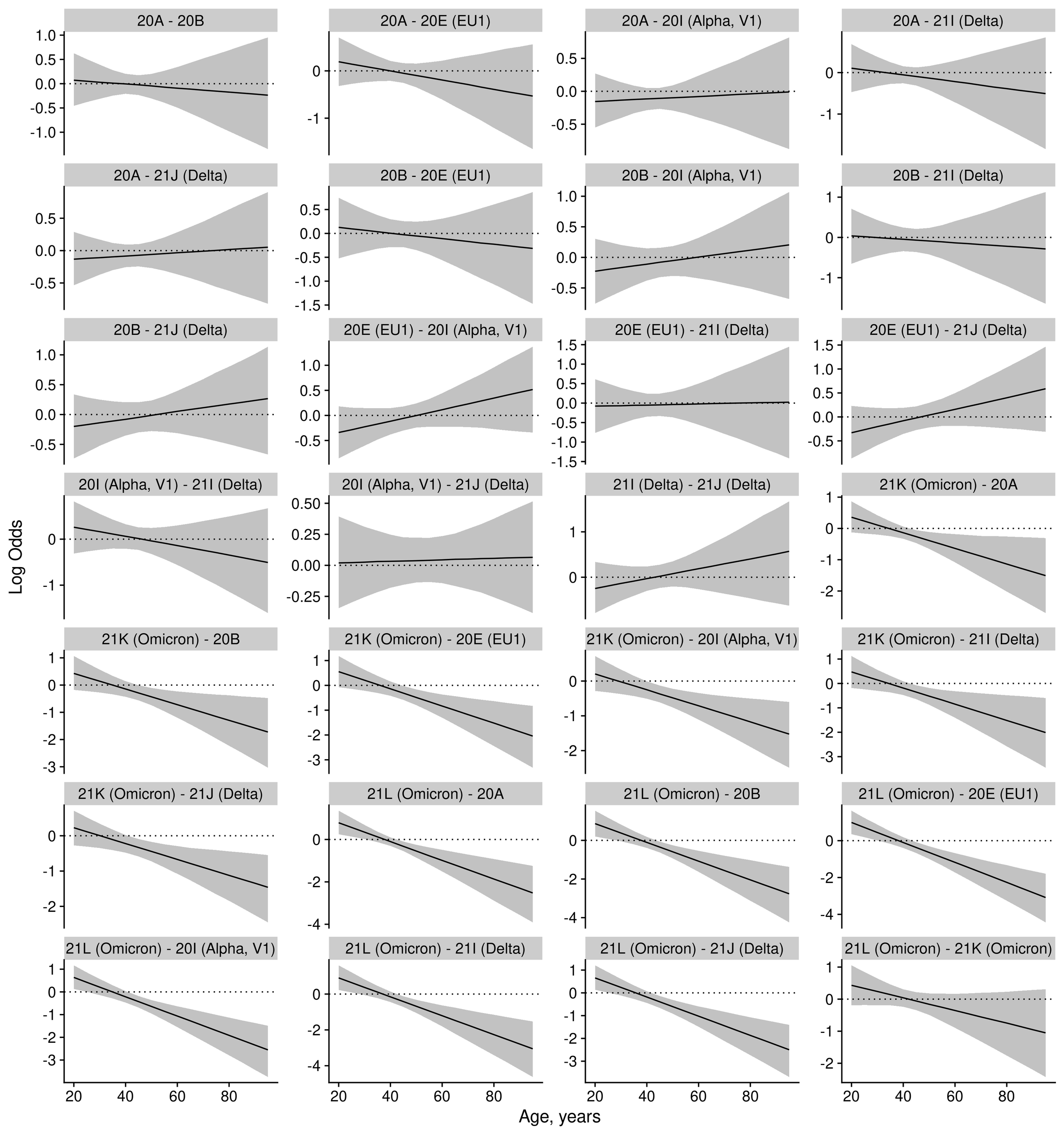

Supplement: S7 Fig — The odds ratios of hospitalization among the unvaccinated population, pairwise comparisons of the SARS-CoV-2 clades, associated with age, averaged over both sexes. Posterior distributions of the estimated marginal means were obtained from the same model as in Fig 5. The line denotes the model’s best estimate and the ribbon denotes a 95% credible interval. The dotted line denotes the log odds ratio = 0. The model summary is presented in Table 4 in S2 Appendix. (TIF) [file pone.0303176.s008.tif]

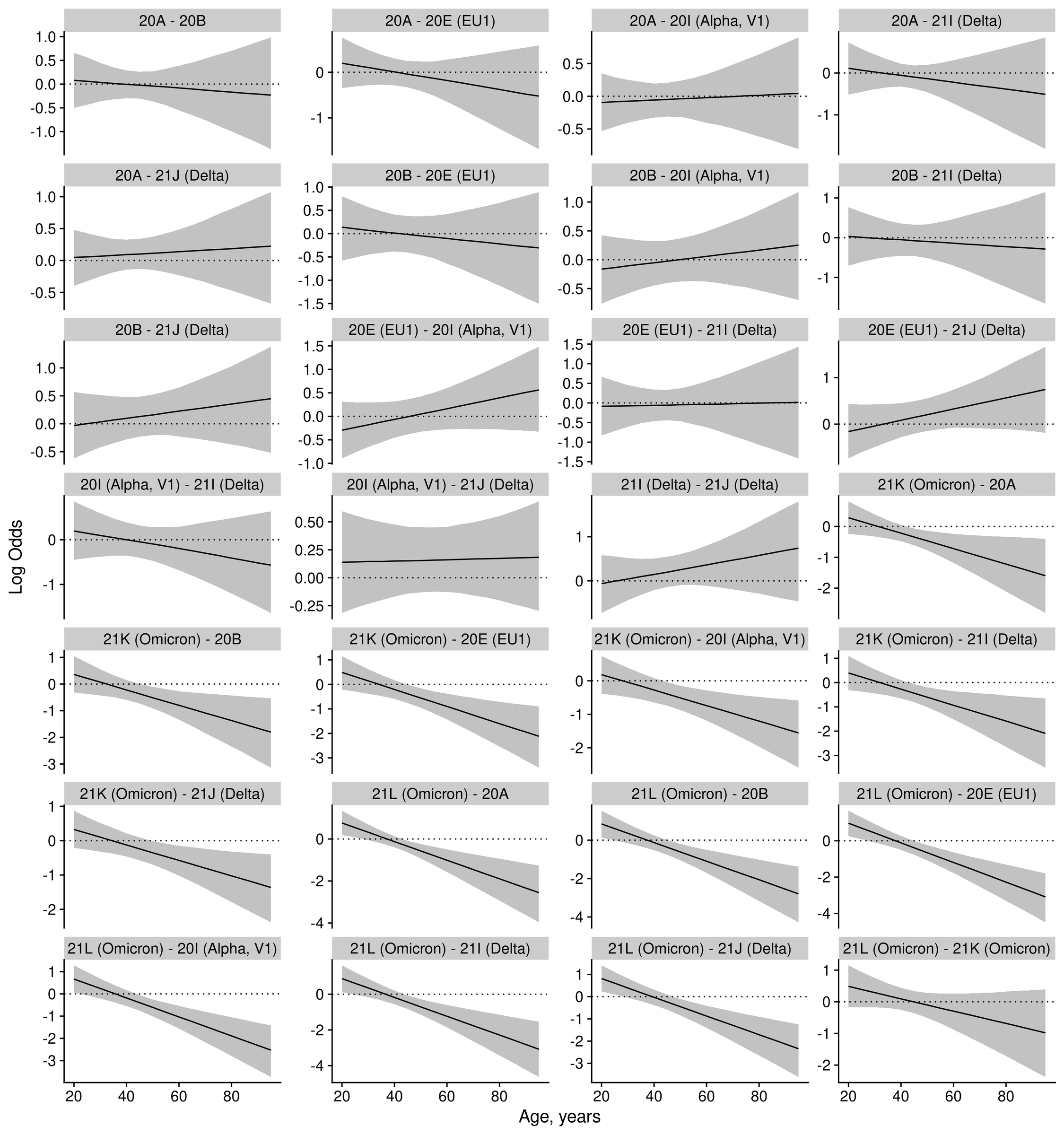

Supplement: S8 Fig — The odds ratios of hospitalization among the vaccinated population, pairwise comparisons of the SARS-CoV-2 clades, associated with age, averaged over both sexes. Posterior distributions of the estimated marginal means were obtained from the same model as in Fig 5. The line denotes the model’s best estimate and the ribbon denotes a 95% credible interval. The dotted line denotes the log odds ratio = 0. The model summary is presented in Table 4 in S2 Appendix. (TIF) [file pone.0303176.s009.tif]
