# Supplementary material for: SARS-CoV-2 clade dynamics and their associations with hospitalisations during the first two years of the COVID-19 pandemic
Source: PLoS One. 2024 May 10;19(5):e0303176. doi: 10.1371/journal.pone.0303176 (PMC11086870; doi:10.1371/journal.pone.0303176)
Supplement: S1 Appendix — (DOCX) [file pone.0303176.s010.docx]

# **Methods**

## SARS-CoV-2 sample collection

Residual SARS-CoV-2 positive nucleic acid samples were obtained from SARS-CoV-2 PCR testing laboratories. The majority of samples (91%) originated from SYNLAB Eesti OÜ which performed >90% of confirmatory SARS-CoV-2 tests in Estonia. The rest of the samples were collected from hospitals’ and Estonian Health Board’s laboratories. Hospitalization was not known during the selection of samples for sequencing, including samples from hospitals. The latter consisted of cases hospitalized for symptomatic COVID-19, those hospitalized for a non-COVID-19 reason, with incidental SARS-CoV-2 infection, and those screened in hospitals.

## Definitions

A fully vaccinated case was defined as a person who received his/her second dose of Pfizer, Moderna, or AstraZeneca, or first dose of Janssen/J&J > 14 days before testing PCR-positivity for SARS-CoV-2. Real-time RT-PCR cycle threshold (Cq) values were available as individual Cq values of ORF1ab, S, and N gene before 01 January 2022, and thereafter as a combined Cq value when TaqPath™ COVID-19 CE-IVD RT-PCR Kit (ThermoFisher Scientific) was switched to TaqPath™ COVID-19, FluA/B, RSV Combo Kit (ThermoFisher Scientific) for SARS-CoV-2 confirmatory testing in SYNLAB Eesti OÜ. The start and end of the VOC wave were defined as a week when the lower or upper bound, respectively, of the 95% confidence interval, calculated using the Clopper-Pearson method, crossed the 50% threshold.

## SARS-CoV-2 sequencing by the European Centre for Disease Prevention and Control (ECDC)

The sequencing of 10,800 (39%) samples was brokered by the ECDC (Eurofins Genomics Germany GmbH, Germany) (Fig 1). For that, the specimens were selected by Cq such that samples with Cq <= 30 were sequenced. The reverse transcription and amplification of SARS-CoV-2 RNA were carried out using the laboratory in-house method similar to the ARTIC primers (consisting of over 200 primer pairs, covering the full 29.9 kb viral genome) and sequenced by Illumina NovaSeq TM 6000 using paired-end 150 bp reads (https://eurofinsgenomics.eu/en/next-generation-sequencing/applications/artic-sars-cov-2-rna-seq/).

## Analysis of 2.5 kb amplicon sequences

The analyses of sequences of 2.5 kb amplicons were carried out by an in-house developed workflow (https://github.com/hivlab/sarscov2-variation). Briefly, the workflow aligns quality trimmed PE short reads to SARS-CoV-2 Wuhan-Hu-1 (NC_045512.2) reference genome, then calls variations, generates consensus sequence, and produces a quality control report. Reads were quality trimmed with BBDuk using the following options “minlen=50 maq=20 qtrim=r trimq=10 ktrim=r k=23 mink=11 hdist=1 tbo tpe ref=adapters ftm=5 ordered”. The quality trimming step removes Illumina Truseq and Nextera adapters, as well as other unknown adapters, based on read pair overlap detection (“tbo” option). The artefact filtering step removes all reads that have a 31-mer match to PhiX and other artefacts. After quality trimming, sequences were corrected using BBMerge based on sequence overlap using the following options “ecco mix vstrict ordered”. Next, sequences were corrected with Tadpole using options “mode=correct k=50 ordered”. Trimmed and quality corrected reads were aligned to reference genome using BBMap with options “append maxindel=200 usemodulo slow k=12”. Nucleotide variations and indels were called with Callvariants using options “minallelefraction=0.05 strandedcov”. Coverage statistics were obtained using Pileup. Variations were quality filtered with vcffilter from vcflib [[1](http://localhost:5607/?capabilities=1&host=http%3A%2F%2F127.0.0.1%3A11679#ref-Garrison2021)] using options “AF > 0.7 & QUAL > 20”. Consensus sequences were generated using a custom workflow script using samtools, bcftools [[2](http://localhost:5607/?capabilities=1&host=http%3A%2F%2F127.0.0.1%3A11679#ref-Danecek2021)], and bedtools [[3](http://localhost:5607/?capabilities=1&host=http%3A%2F%2F127.0.0.1%3A11679#ref-Quinlan2010)]. Functional annotation and formatting of variants were done using snpeff [[4](http://localhost:5607/?capabilities=1&host=http%3A%2F%2F127.0.0.1%3A11679#ref-Cingolani2012)] and snpsift [[5](http://localhost:5607/?capabilities=1&host=http%3A%2F%2F127.0.0.1%3A11679#ref-Ruden2012)] packages, respectively. QC report was generated using MultiQC [[6](http://localhost:5607/?capabilities=1&host=http%3A%2F%2F127.0.0.1%3A11679#ref-Ewels2016)]. BBDuk, BBMerge [[7](http://localhost:5607/?capabilities=1&host=http%3A%2F%2F127.0.0.1%3A11679#ref-Bushnell2017)], Tadpole, BBMap, Callvariants, and Pileup are from the BBTools package (https://sourceforge.net/projects/bbmap/). Workflow execution was managed using Snakemake [[8](http://localhost:5607/?capabilities=1&host=http%3A%2F%2F127.0.0.1%3A11679#ref-Moelder2021)]. Human reads were removed from raw sequence libraries by mapping the reads to the human reference genome (HumanG1Kv37) with BBMap.

## Analysis of ARTIC protocol v4 primer sequences

ARTIC protocol v4 primer amplicon sequences were analyzed using COVID-19: variation analysis on ARTIC PE data (v0.5) Galaxy workflow (https://workflowhub.eu/workflows/110). Briefly, paired-end sequences were aligned using BWA-mem [[9](http://localhost:5607/?capabilities=1&host=http%3A%2F%2F127.0.0.1%3A11679#ref-Li2013)] and variant calling was done with LoFreq [[10](http://localhost:5607/?capabilities=1&host=http%3A%2F%2F127.0.0.1%3A11679#ref-Wilm2012)], amplicon primer sequences were trimmed with iVar [[11](http://localhost:5607/?capabilities=1&host=http%3A%2F%2F127.0.0.1%3A11679#ref-Grubaugh2019)]. iVar was also used to identify amplicons affected by primer-binding site mutations and reads derived from affected amplicons were excluded from allele-frequency calculation of other variants. Consensus sequences were constructed with COVID-19: consensus construction (v0.3) Galaxy workflow (https://workflowhub.eu/workflows/138). Variant calling results were converted to tabular reports using COVID-19: variation analysis reporting (v0.2) Galaxy workflow (https://workflowhub.eu/workflows/109). Lineage and clade assignment for the SARS-CoV-2 genome consensus sequences was performed using the Pangolin (https://cov-lineages.org/resources/pangolin.html) and NextClade (https://clades.nextstrain.org/ ) tools, respectively. Human reads were removed from raw sequence libraries by mapping the reads to the human reference genome (Hg38) with BWA-mem (https://workflowhub.eu/workflows/4).

# **References**

1. Garrison E, Kronenberg ZN, Dawson ET, Pedersen BS, Prins P. Vcflib and tools for processing the VCF variant call format. bioRxiv. 2021. doi:[10.1101/2021.05.21.445151](https://doi.org/10.1101/2021.05.21.445151)

2. Danecek P, Bonfield JK, Liddle J, Marshall J, Ohan V, Pollard MO, et al. Twelve years of SAMtools and BCFtools. GigaScience. 2021;10. doi:[10.1093/gigascience/giab008](https://doi.org/10.1093/gigascience/giab008)

3. Quinlan AR, Hall IM. BEDTools: a flexible suite of utilities for comparing genomic features. Bioinformatics. 2010;26: 841–842. doi:[10.1093/bioinformatics/btq033](https://doi.org/10.1093/bioinformatics/btq033)

4. Cingolani P, Platts A, Wang LL, Coon M, Nguyen T, Wang L, et al. A program for annotating and predicting the effects of single nucleotide polymorphisms, SnpEff. Fly. 2012;6: 80–92. doi:[10.4161/fly.19695](https://doi.org/10.4161/fly.19695)

5. Ruden D, Cingolani P, Patel V, Coon M, Nguyen T, Land S, et al. Using drosophila melanogaster as a model for genotoxic chemical mutational studies with a new program, SnpSift. Frontiers in Genetics. 2012;3. doi:[10.3389/fgene.2012.00035](https://doi.org/10.3389/fgene.2012.00035)

6. Ewels P, Magnusson M, Lundin S, Käller M. MultiQC: summarize analysis results for multiple tools and samples in a single report. Bioinformatics. 2016;32: 3047–3048. doi:[10.1093/bioinformatics/btw354](https://doi.org/10.1093/bioinformatics/btw354)

7. Bushnell JAS Brian AND Rood. BBMerge – accurate paired shotgun read merging via overlap. PLOS ONE. 2017;12: 1–15. doi:[10.1371/journal.pone.0185056](https://doi.org/10.1371/journal.pone.0185056)

8. Mölder F, Jablonski KP, Letcher B, Hall MB, Tomkins-Tinch CH, Sochat V, et al. Sustainable data analysis with Snakemake. F1000Research. 2021;10: 33.

9. Li H. Aligning sequence reads, clone sequences and assembly contigs with BWA-MEM. arXiv; 2013. doi:[10.48550/ARXIV.1303.3997](https://doi.org/10.48550/ARXIV.1303.3997)

10. Wilm A, Aw PPK, Bertrand D, Yeo GHT, Ong SH, Wong CH, et al. LoFreq: A sequence-quality aware, ultra-sensitive variant caller for uncovering cell-population heterogeneity from high-throughput sequencing datasets. Nucleic acids research. 2012;40: 11189–201.

11. Grubaugh ND, Gangavarapu K, Quick J, Matteson NL, De Jesus JG, Main BJ, et al. An amplicon-based sequencing framework for accurately measuring intrahost virus diversity using PrimalSeq and iVar. Genome Biology. 2019;20: 8. doi:[10.1186/s13059-018-1618-7](https://doi.org/10.1186/s13059-018-1618-7)
